# Utility of Chemotherapy for Metastatic Melanoma in the Contemporary Era and Experience with Modified Vinblastine–Cisplatin–Temozolomide at a Tertiary Care Center

**DOI:** 10.3390/biomedicines14102274

**Published:** 2026-10-08

**Authors:** Woo Kyung Lee Doolittle, Rohit Rao, Malena Johnson, Felicia Tejawinata, Akihiro Yoshida, Luke Daniel Rothermel, Ankit Mangla

**Affiliations:** 1 Department of Hematology and Oncology, University Hospitals Seidman Cancer Center, Cleveland, OH 44106, USA; wookyung.doolittle@uhhospitals.org (W.K.L.D.); rohit.rao@uhhospitals.org (R.R.); 2 Department of Hematology and Oncology, Case Western Reserve University School of Medicine, Cleveland, OH 44106, USA; 3 Case Western Reserve University School of Medicine, Cleveland, OH 44106, USA; mbj43@case.edu (M.J.); fit4@case.edu (F.T.); 4 Department of Dermatology, Case Western Reserve University School of Medicine, Cleveland, OH 44106, USA; axy234@case.edu; 5 Case Comprehensive Cancer Center, Cleveland, OH 44106, USA; luke.rothermel@uhhospitals.org; 6 Department of Surgical Oncology, University Hospitals Cleveland Medical Center, Cleveland, OH 44106, USA

**Keywords:** chemotherapy, vinblastine, cisplatin, temozolomide, melanoma, brain metastasis, trial ineligibility, social barriers

## Abstract

**Purpose:** Chemotherapy can be a viable option for patients with relapsed-refractory melanoma (RRM) who do not have access to advanced treatments or clinical trials. We report our experience with a modified Vinblastine–Cisplatin–Temozolomide (mVCT) regimen in patients with RRM and barriers to their enrollment in clinical trials. **Methods:** We conducted a retrospective review of patients with RRM who received mVCT. The primary endpoint was overall response rate (ORR) following the last cycle. Secondary endpoints included the rate of complete response (CR), partial response (PR), stable disease (SD), clinical benefit rate (CBR), progression-free survival (PFS), intracranial (IC) PFS, (IC) CBR, (IC) RR, and toxicity. **Results:** Ten patients received mVCT for a median of 4.5 cycles (IQR: 3–6). Median follow-up was 20 months (IQR: 10.3–30.3). The ORR was 50% (CR-1, PR-4) and CBR was 70%. The median PFS was 7 months (IQR: 1.5–9.25). Among eight patients with brain metastasis, six underwent gamma-knife radiosurgery (GKRS); three achieved PR, and three had SD. Two patients without GKRS achieved CR, leading to an ICRR of 62.5% and an ICCBR of 100%. The median ICPFS was 7.5 months (IQR: 6–9.75). Median OS was 22 months (95% CI: 15.6–32.4). Four patients experienced grade ≥ 3 toxicities (fatigue in two, myelosuppression in two); two required dose reduction and one discontinued treatment. Two patients with PD later enrolled in phase 1 clinical trials. Brain metastasis (80%), immunotherapy-related toxicity (60%), acral/mucosal melanoma (50%), and social barriers (40%) were the major reasons for trial ineligibility. **Conclusions:** mVCT is an effective and well-tolerated regimen for patients with RRM and can serve as a bridge while they await access to advanced treatments or a clinical trial.

## 1. Introduction

Melanoma accounts for 1–4% of all skin cancers yet is the deadliest of all skin cancers [1]. Despite an increase in incidence, melanoma-related deaths have experienced a sharp decline [2]. American Cancer Society lists melanoma as the fifth most common cancer in 2026 but does not count it among the top 10 causes of cancer-related death [3]. Whilst treatment for early-stage melanoma is rooted in wide local excision (WLE), with or without sentinel node biopsy (SLNB), treatment for advanced melanoma has undergone several advancements in the last decade [2,4,5,6,7,8]. Immune checkpoint inhibitor (ICI) therapy has become an integral part of melanoma therapeutics used in neoadjuvant, adjuvant, and metastatic settings. Likewise, in patients with melanoma expressing the BRAF V600E/K mutation, BRAF (B-Raf proto-oncogene, serine/threonine kinase) and MEK (mitogen-activated extracellular signal-regulated kinase) inhibitors (BRAF/MEK-I) can be useful in sequence with checkpoint inhibitors [2,4]. In patients with resistance to both, tumor-infiltrating lymphocyte (TIL) therapy is an option, although access to this resource-intensive, multidisciplinary treatment remains privileged [2,4]. Oncolytic viral therapies, with or without ICI, are used in a select few patients under special circumstances (such as in-transit disease, primary resistance to ICI monotherapy, etc.) [9]. When available, national guidelines recommend enrollment in clinical trials, regardless of standard treatment options [4]. Several new agents, such as chimeric antigen receptor (CAR) T-cell therapy, T-cell receptor (TCR)-based therapies, and intratumoral cytokines, have been gaining significant traction in the treatment paradigm for patients with advanced melanoma [10,11,12,13]. A recent spate of tissue-agnostic approvals for several agents has created additional options for patients with melanoma when the tumor expresses these mutations (like NTRK fusions, ERBB2 expression, and RET Gene fusions) [14].

Chemotherapy has fallen out of favor for patients with advanced melanoma. Poor tolerance, inferior responses, and non-durability of response are the known downsides of chemotherapy [15,16,17]. However, chemotherapy is a readily accessible treatment available to patients with metastatic melanoma, especially those who are unable to participate in clinical trials and have poor access to TIL therapy. The unavailability of clinical trials at the treating institution, untreated or symptomatic brain metastasis, diagnosis of autoimmune disease or transplant status requiring immunosuppression, and a previous history of grade 3/4 immunotherapy-related adverse events (IRAEs) are among the most common causes of ineligibility for clinical trials [18,19,20,21]. For similar reasons, many patients will also not be able to receive advanced treatments like TIL therapy or intratumoral oncolytic viral or cytokine therapy [9,13]. The presence of symptomatic autoimmune diseases requiring immunosuppression, solid organ or bone marrow transplant, and symptomatic brain metastasis requiring use of corticosteroids are usual barriers to successful use of any immunotherapy. In this manuscript, we present our experience with a modified version of Vinblastine–Cisplatin–Temozolomide (mVCT) chemotherapy regimen in patients with metastatic melanoma. We will also present the reasons and scenarios in which such patients may benefit from this chemotherapy regimen. This manuscript presents a tolerable, accessible regimen for patients while they wait for a better, preferably immunotherapy-based, option.

## 2. Materials and Methods

This single-center, retrospective, observational review was conducted at the University Hospitals Seidman Cancer Center in Cleveland for patients with relapsed/refractory melanoma (RRM) between 1 January 2019 and 30 June 2024. We defined RRM as melanoma progression despite dual checkpoint inhibition and BRAF-MEK-I (for BRAF V600E/K-mutant melanoma). We included patients who were at least 18 years old, had pathologically confirmed metastatic sites, had discontinued prior treatment because of disease progression or grade 3/4 toxicities, and received at least 1 cycle of mVCT chemotherapy because they were ineligible for a clinical trial. We excluded patients with inadequate medical records. We collected demographic information, tumor type/stage at first diagnosis and at the start of mVCT chemotherapy, previous treatments, reasons for trial ineligibility, and toxicities from the mVCT regimen. The University Hospital’s Institutional Review Board approved this study (protocol code STUDY20240271), and it was conducted in accordance with the ethical standards of the 1964 Declaration of Helsinki and all relevant United States Health Insurance Portability and Accountability Act regulations.

### 2.1. Treatment Protocol

The treatment protocol was as follows: cisplatin 20 mg/m^2^ and vinblastine 1.5 mg/m^2^ given intravenously (IV) on days 1, 2, and 3. Temozolomide (TMZ) was given orally at 125 mg/m^2^ (rounded off to the nearest capsule strength) on days 1, 2, 3, 4, and 5. Premedications included fosaprepitant 150 mg IV on day 1, palonosetron 0.25 mg IV on days 1 and 3, diphenhydramine 50 mg PO on days 1–3, and dexamethasone 12 mg IV on days 1–3. Pre- and post-hydration with normal saline was administered on days of cisplatin administration. We gave prophylactic granulocyte-monocyte colony-stimulating factor (GM-CSF) to all patients. We monitored electrolytes and replaced them as needed (since cisplatin can cause renal wasting of magnesium and potassium). With TMZ, we also gave Pneumocystis jirovecii prophylaxis with sulfamethoxazole (800 mg) and trimethoprim (160 mg) (also called Bactrim double strength), one tablet by mouth every Monday, Wednesday, and Friday. We prescribed supportive medications for nausea (ondansetron and olanzapine preferred) according to national guidelines. The treatment was planned to continue until progression or intolerance.

We planned dose reductions in individual drugs or all drugs depending on the adverse event. For instance, if excessive myelosuppression was noted, we reduced the TMZ dose by decreasing the number of administration days. Likewise, if excessive nausea and vomiting were observed, we planned to reduce the dose of cisplatin. In patients with severe fatigue or multiple side effects, we sequentially reduced the dose of all drugs by 30%.

### 2.2. Difference in Doses from the Original Protocol and Rationale for Reduced Dosing

The Hellenic Cooperative Oncology Group (HECOG) protocol gave cisplatin at 30 mg/m^2^ (days 1–3), vinblastine at 2 mg/m^2^ (days 1–3), and TMZ at 150 mg/m^2^ from Days 1–5. However, none of the patients in this trial had received prior treatment or had brain metastasis. Patients in our cohort had received previous immunotherapy and/or targeted therapy (with BRAF and MEK inhibitors), and many of those with brain metastasis had also received stereotactic radiation (Table 1). Our treatment goal was palliation and to prevent progression until a trial became available. We also aimed to minimize the risk of organ dysfunction (kidney or liver dysfunction, or excessive myelosuppression) to allow patients to quickly pivot to clinical trials if one became available. Hence, we reduced the dose of all three drugs to increase tolerance while maintaining efficacy.

**Figure 1:**
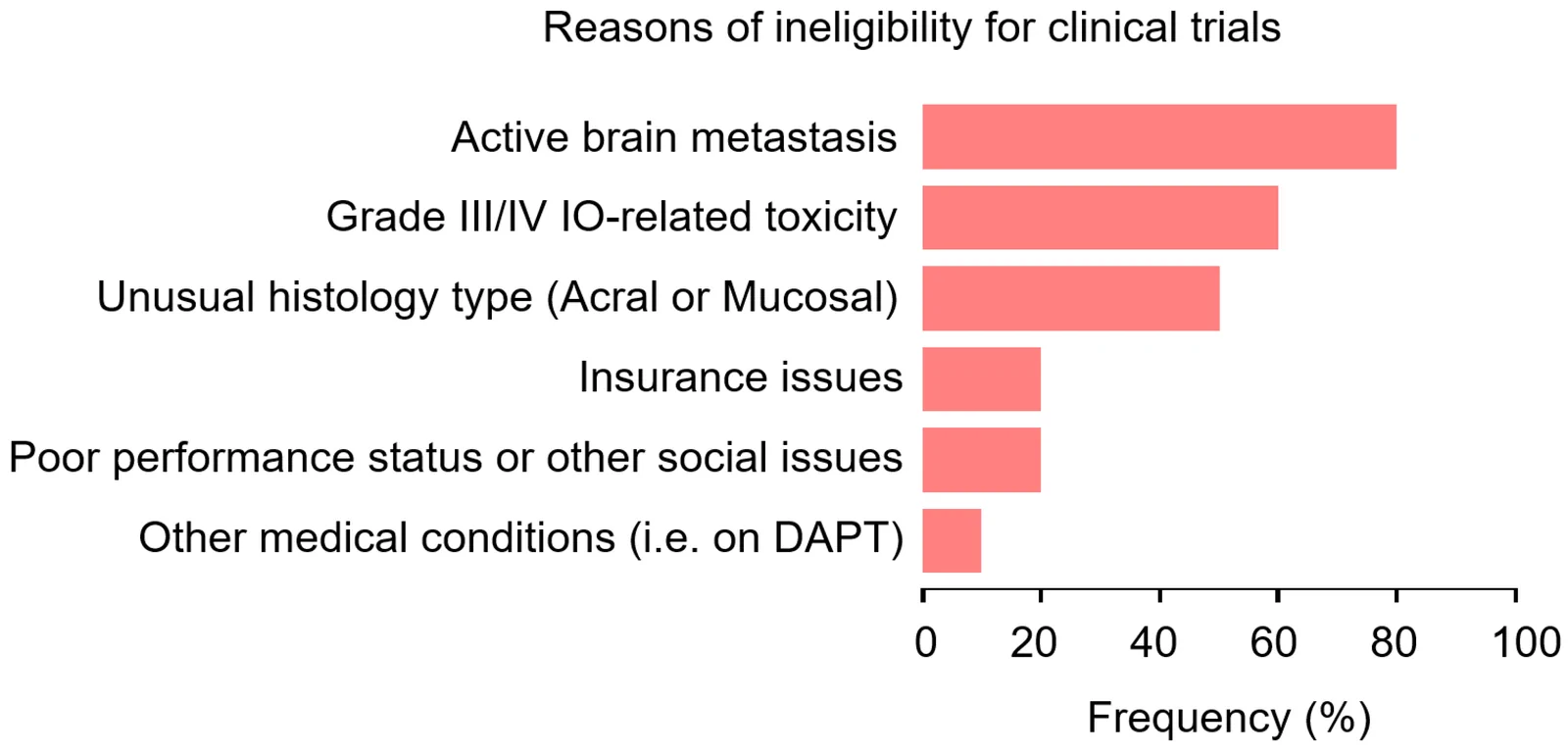
The bar graphs showing the frequencies of the various reasons for trial ineligibility.

We reviewed ten patients with the above criteria who received the mVCT regimen. We identified various reasons why these patients could not participate in clinical trials (Figure 1). Next, tumor response was reviewed by radiologists and then cross-checked by all listed investigators according to RECIST 1.1. Complete response (CR), partial response (PR), stable disease (SD), and progressive disease (PD) were recorded. We defined overall response rate (ORR = CR + PR) following the last cycle as the primary endpoint. For secondary endpoints, we assessed progression-free survival (PFS), clinical benefit rate (CBR = CR + PR + SD), intracranial response rate (ICRR), intracranial clinical benefit rate (ICCBR), intracranial progression-free survival (ICPFS), and overall survival (OS). Using a Kaplan–Meier estimator, we measured PFS from the start of mVCT to documented PD; patients without PD were censored at their last progression-free tumor assessment before starting the next line of treatment. OS was calculated from the start of mVCT to death from any cause or censored at last known contact. We also reviewed toxicities after the last cycle of mVCT and whether these required dose reduction or discontinuation during the course. We graded toxicities according to the Common Terminology Criteria for Adverse Events version 5.0. Finally, we tracked the tumor response over the course and the best tumor response in non-brain lesions and brain lesions. We described continuous variables as mean ± standard deviation or median with interquartile range and reported categorical variables as the number of cases with relative frequencies. We used IBM SPSS 23 and GraphPad Prism 10.1.1 for statistical analysis and data visualization.

## 3. Results

### 3.1. Baseline Characteristics of the Study Population

The mean age of the study population was 60.2 ± 14.8 years, with six men and nine white patients (Table 1). Five patients had non-acral cutaneous melanoma, three had acral, and two had mucosal melanoma. All patients previously received ICI (anti-PD1 (programmed cell death protein 1) and/or anti-CTLA4 (cytotoxic T-lymphocyte-associated protein 4), and four of them also had other treatments, including targeted therapies (BRAF-MEK-I, PARP (poly (ADP-ribose) polymerase) inhibitor, or multiple tyrosine kinase inhibitors), chemotherapies besides mVCT, and other types of immunotherapies (interferon, peg-interleukin-12, or TVEC (talimogene laherparepvec) indicating their relapsed refractory disease. Upon their disease progression prior to the mVCT, nine patients had metastatic disease, with eight having brain metastasis (M1d) and one having lung metastasis (M1b). The remaining patient had locally advanced, unresectable stage 3 disease. Among those with brain metastasis, six patients had previously received gamma-knife radiosurgery (GKRS).

### 3.2. Reasons for Trial Ineligibility

The various reasons for trial ineligibility are shown in Figure 1. The most common reasons were untreated brain metastasis (8 out of 10, 80%) and previous grade 3 or 4 ICI-related toxicities (6 out of 10, 60%), such as hepatitis, colitis, pneumonitis, dermatitis, and hematologic and neurologic toxicities, followed by the rarer melanoma subtypes (5 out of 10, 50%), including acral and mucosal melanoma. In addition, social issues were significant barriers, including insurance issues (*n* = 2), poor performance status (*n* = 2), and a long commute leading to transportation issues (*n* = 2). One patient could not participate in a trial due to being on dual antiplatelet therapy essential for her cardiovascular condition.

### 3.3. mVCT as an Effective Therapeutic Option for RRM

**Figure 2:**
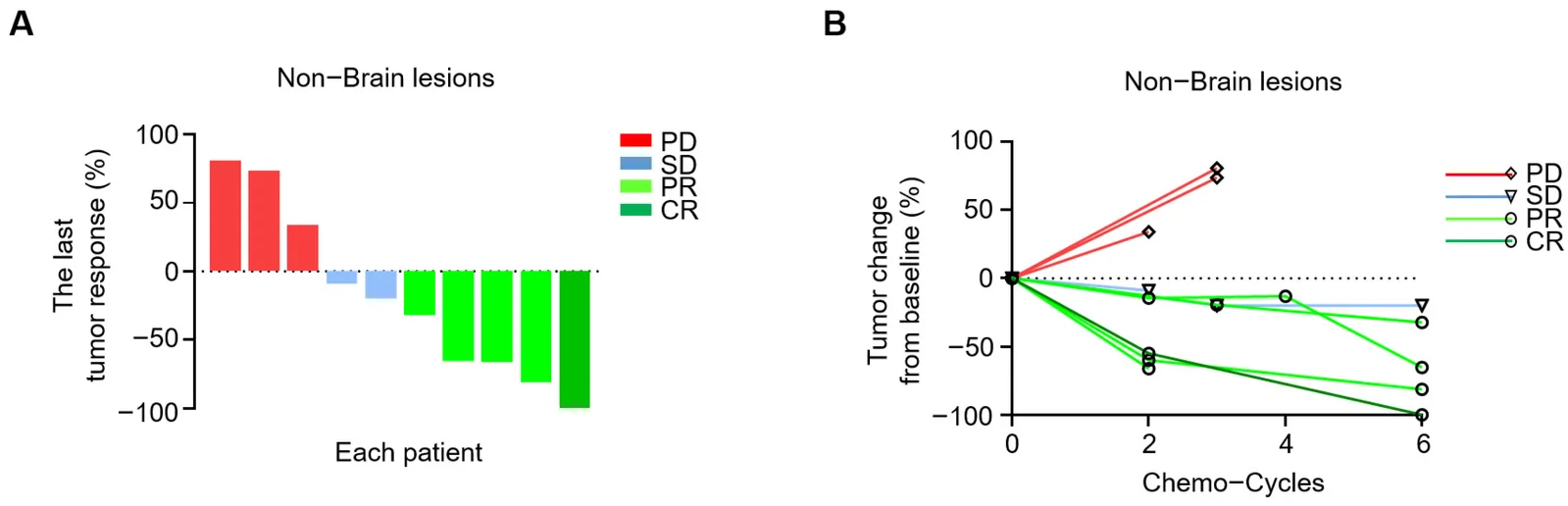
A waterfall plot (**A**) and a spider plot (**B**) showing the best tumor response and tumor burden of non-brain lesions over the course of mVCT.

**Figure 3:**
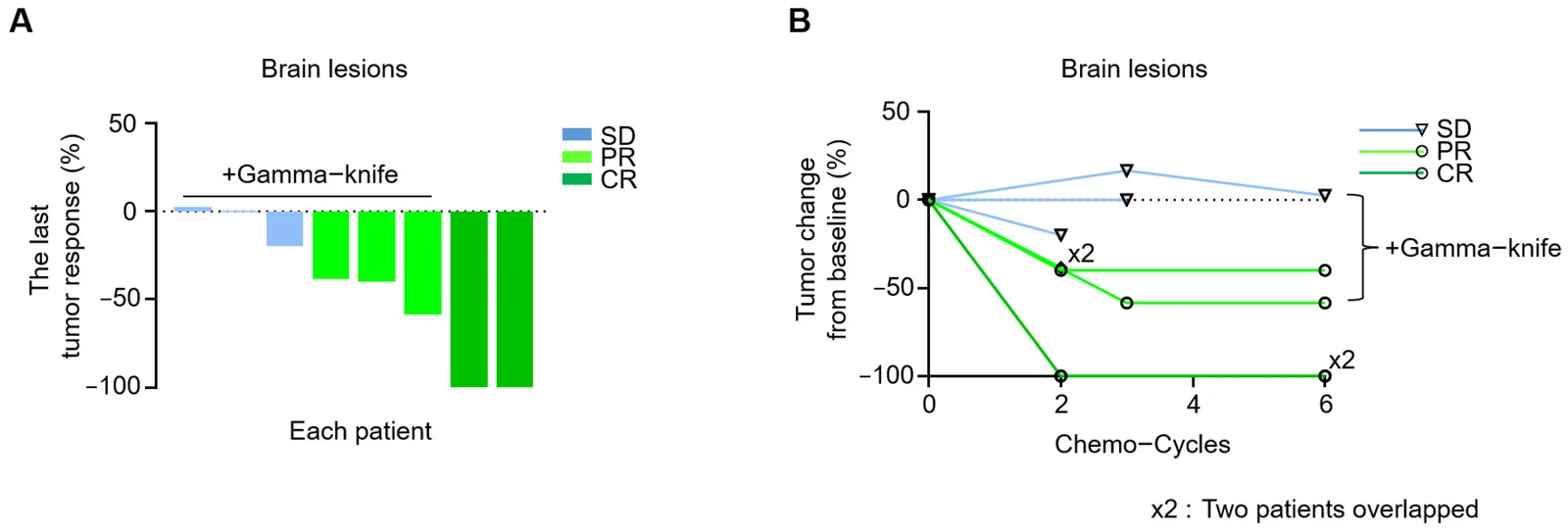
A waterfall plot (**A**) and a spider plot (**B**) showing the best tumor response and tumor burden of brain lesions over the course of mVCT.

The study population received the mVCT regimen for a median of 4.5 cycles (Interquartile range (IQR): 3–6; Table 1). Median follow-up duration after starting mVCT was 20 months (IQR: 10.3–30.3). Following the last cycle, 1, 4, and 2 patients achieved CR, PR, and SD, respectively, with an ORR of 50% and a CBR of 70% (Figure 2A). These patients showed durable tumor response over the course of treatment (Figure 2B) with a median PFS of 7 months (IQR: 1.5–9.25; Table 1). Among the eight patients with brain metastases, two who did not receive GKRS achieved CR with mVCT. Of the six patients with prior GKRS, three maintained PR, and three had SD with mVCT, resulting in an ICRR of 62.5% and an ICCBR of 100% (Table 1 and Figure 3A). Similar to extracranial responses, brain lesions showed durable response over time (Figure 3B) with a median ICPFS of 7.5 months (IQR: 6–9.75; Table 1).

### 3.4. Tolerability of the mVCT Regimen

The most common toxicity of the mVCT was fatigue, occurring in seven patients; two developed grade ≥ 3 fatigue, leading to dose reduction (patient 2) and discontinuation (patient 10) (Table 1). Only two patients developed significant myelosuppression, one requiring a dose reduction (patient 5).

### 3.5. Post-mVCT Treatment Course and Overall Survival

Table 2 summarizes post-mVCT treatment courses. Patients 7 and 9, both with acral melanoma and PD on mVCT, later enrolled in phase 1 trials—TIL therapy and a pan-RAF inhibitor, respectively—once available. Patient 7 received TIL therapy at an outside institution but developed ICI-related encephalitis, followed by PD and death. Patient 9 responded to the pan-RAF inhibitor for nearly 6 months before the trial was halted for dose-limiting toxicities; a planned TIL harvest subsequently failed, and he died of PD.

**Figure 4:**
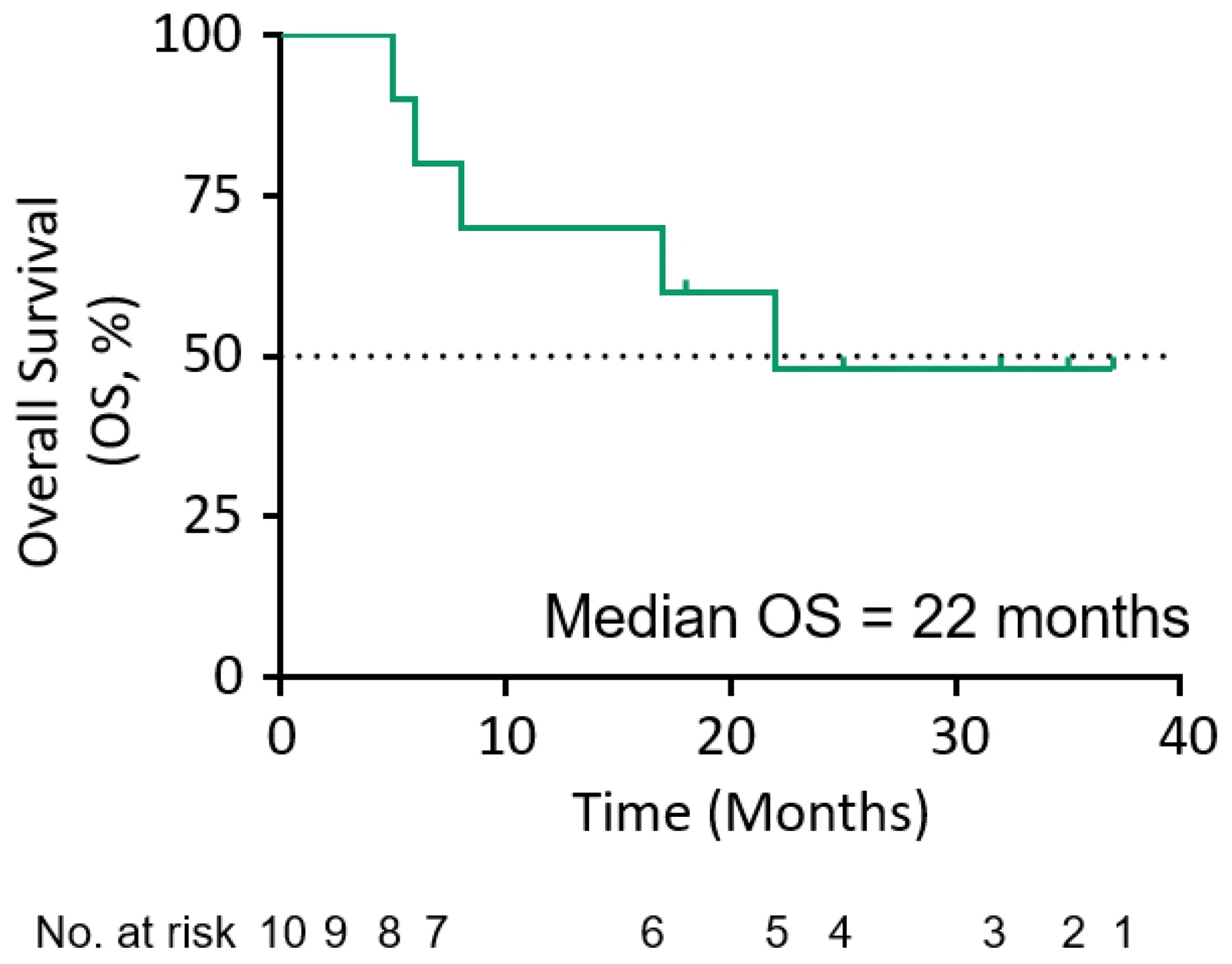
A Kaplan–Meier survival curve showing overall survival of the study population.

Of note, Patient 3, who had a strong response to mVCT (PR extracranially, CR intracranially), was rechallenged with 6 additional cycles of mVCT after developing grade 3 pneumonitis on pembrolizumab, achieving SD. She was then switched to olaparib for a Homologous Recombination Deficiency (HRD)-positive signature on genomic sequencing. However, she could not tolerate olaparib and recently received TIL therapy, with stable, brain-metastasis-free disease. In addition, Patient 4 completed 2 years of pembrolizumab after 6 cycles of mVCT and remains in CR on surveillance. Patient 10 tolerated 10 cycles of TMZ combined with pembrolizumab, then pembrolizumab monotherapy, and remains in CR, with serial skin biopsies showing melanophage infiltration consistent with regressed melanoma. At the last follow-up, 5 patients were alive (Table 2) and median OS was 22 months (95% CI: 15.6–32.4, Figure 4).

## 4. Discussion

RRM is a devastating disease with a 5-year survival rate of 16–18% [22]. While ICI-based regimens are the mainstay of treatment, about 40% of patients show innate resistance to immunotherapy [23]. In patients with BRAF V600E/K-mutated melanoma, BRAF-MEK-I provides a viable alternative [24,25,26]. The median duration of response with BRAF-MEK-I in treatment-naïve patients is 18.6 months [27]. However, in patients with immunotherapy (IO)-refractory melanoma, BRAF-MEK-I adds a median of 9.8 months of overall survival (OS) [28]. The USFDA (United States Food and Drug Administration) recently approved Lifileucel^®^, the first TIL therapy, for patients with melanoma that has progressed on anti-PD1 monotherapy and on BRAF/MEK-I (in BRAF V600E/K-mutated melanoma). TIL therapy is resource-intensive, associated with multiple challenges, and requires careful patient selection [29]. Intralesional oncolytic virus therapy, TVEC (Talimogene Laherparepvec), is an FDA-approved intratumoral therapy that provides modest benefits in patients with anti-PD1-resistant melanoma [9]. For patients with immunotherapy-resistant melanoma, clinical trials offer the best treatment option. However, there are several challenges in clinical trial enrollment.

### 4.1. Is Chemotherapy a Viable Option for Patients in the Contemporary Era?

The floodgates for immunotherapy approval have opened since the first approval of ipilimumab in 2011 for metastatic melanoma. In the age of immunotherapy and targeted therapy, chemotherapy has taken a backseat in the treatment paradigm of patients with metastatic melanoma. However, in the contemporary era, the impact of chemotherapy on the prognosis of patients with RRM is not clearly understood. In our study, we examined the efficacy and safety of mVCT, a regimen utilized in the pre-IO era, as a salvage therapy for trial-ineligible patients, including those with brain metastases. We found this regimen safe and active in patients with RRM, and it may serve as an alternative until a clinical trial or advanced treatments become available.

In patients with ICI-refractory metastatic melanoma, dacarbazine (DTIC) monotherapy, fotemustine, or DTIC in combination with other cytotoxic agents have been reported to have a median PFS of 2.4–5.4 months [30,31]. In a cohort of patients with predominantly ICI-refractory, BRAF wild-type melanoma, the median PFS with chemotherapy was reported to be 1.6 months [32]. Chemotherapy combinations with biologics have also been tested in the salvage setting. Treatment of RRM with TMZ or Abraxane combined with antiangiogenic therapy has been found to have a median PFS of 3 months [33]. Similarly, a combination of chemotherapy (mostly carboplatin/paclitaxel) with immunotherapy in RRM has had a median event-free survival (EFS) of 7.6 months, extending to 9 months in those with BRAF WT melanoma [34]. On the other hand, rechallenge with MAPK combinations in patients who have progressed on MAPK-targeted therapy has a median PFS of around 5–5.9 months [35,36,37]. In our cohort, we had a 50% overall response rate (ORR) with the mVCT regimen and a median PFS of 7 months. Overall, we find that mVCT shows a comparable or better response to other chemotherapy regimens or MAPK-targeted therapy rechallenge in the salvage setting for patients with metastatic melanoma.

#### 4.1.1. Vinblastine-Cisplatin-Dacarbazine or Vinblastine–Cisplatin–Temozolomide Chemotherapy in Patients with Metastatic Melanoma

Vinblastine-Cisplatin-Dacarbazine (VCD) is the classic cytotoxic regimen devised at MD Anderson Cancer Center (MDACC) [38]. The phase II trial recommended DTIC at 800 mg/m^2^ IV (Day 1), vinblastine 1.6 mg/m^2^/day (Days 1–5), and cisplatin 20 mg/m^2^/day (Days 2–5) [38]. The phase II study showed an ORR of 40%, a median duration of response of 9 months, and a median OS of 9 months [38]. However, randomized trials (like ECOG/Intergroup E3695) have used VCD as the control arm since then, and the results have been underwhelming (ORR 13.8%, median PFS 2.9 months, and median OS 8.7 months) [39]. The substitution of DTIC with TMZ was later attempted in the original bio-chemotherapy regimen from MDACC (by adding interferon-alpha and interleukin-2 to VCD) [40]. This improved median OS from 11.8 months (VCD backbone) to 18.6 months (with VCT backbone) [41]. In addition, this trial showed that substituting TMZ for DTIC affected the dissemination of brain metastasis, had equal activity in non-brain metastasis, and suggested lower toxicity when TMZ was administered orally [41].

The Hellenic Cooperative Group (HECOG) Phase II trial evaluated TMZ plus cisplatin versus TMZ alone [42]. In this trial, TMZ was administered at 200 mg/m^2^/day from Day 1–5 and cisplatin on Day 1 at 75 mg/m^2^. Although the two arms showed no difference in time to progression or OS, the combination arm had significantly higher grade 3 and higher toxicities [42]. Subsequently, HECOG conducted a phase II study of vinblastine, cisplatin, and TMZ based on the positive signal obtained in bio-chemotherapy trials, using VCT as the backbone [41,43,44]. The trial used cisplatin 30 mg/m^2^/day (days 1–3), vinblastine 2 mg/m^2^ (days 1–3), and TMZ 150 mg/m^2^/day (days 1–5) orally. This trial enrolled 35 treatment-naïve patients. The authors reported an ORR of 34% (1 CR and 11 PR), and a CBR of 60% (9 patients had SD). Most patients had grade 3 or higher neutropenia, anemia, and fatigue, and a few had febrile neutropenia. In contrast, the mVCT regimen afforded an ORR of 50% and a CBR of 70%. Only one of ten patients required dose reduction for myelotoxicity, and one for excessive fatigue. None of the patients reported vomiting or hearing loss (cisplatin-induced adverse events). One patient was a high-functioning nonagenarian who tolerated the mVCT regimen and achieved a radiologic PR on functional scans (assessed by PET-CT). Although a dose reduction was needed due to excessive fatigue, the patient received six cycles and had a PFS of 12 months after stopping chemotherapy.

#### 4.1.2. Comparison Between Temozolomide and Dacarbazine

TMZ is often referred to as the oral analog of DTIC [45]. They are both imidazole-tetrazine alkylating agents that require conversion to the same active metabolite, 3-methyl-(triazen-1-yl)imidazole-4-carboximide (MTIC) [46,47]. Both drugs work by methylation of the purine bases of deoxyribonucleic acid (DNA) (O6-guanine, N7-guanine, and N3-adenine) [46,47]. They also share the same resistance pathway, through MGMT (O^6^-methylguanine–DNA methyltransferase), which directly repairs DNA and renders the drug ineffective [48]. Despite these similarities, the drugs differ significantly in pharmacokinetics. TMZ is an oral drug with ~100% bioavailability, while DTIC is an intravenous drug requiring a visit to an infusion center [49]. DTIC requires CYP450-mediated liver metabolism to convert to active metabolites, whereas TMZ undergoes pH-dependent conversion. Hence, DTIC, when administered as 1000 mg/m^2^, is associated with severe nausea, vomiting, and the possibility of hepatic toxicity. TMZ can also cross the blood–brain barrier, thus making it more attractive in patients with melanoma brain metastasis. Thirty percent of patients with melanoma have brain metastasis at the time of presentation, and 80% will have progression at the time of death [50]. In our cohort, the majority of patients had brain metastasis, making TMZ a logical choice over DTIC. A phase III randomized controlled trial (RCT) comparing TMZ with DTIC in advanced melanoma reported a significantly longer PFS and a better quality of life, along with improved physical function, with TMZ [45]. TMZ also has reasonable intracranial activity in patients with melanoma brain metastasis [51].

#### 4.1.3. Therapeutic Challenge from Melanoma Brain Metastasis

Symptomatic melanoma brain metastasis is a therapeutic challenge, especially in patients with RRM. Symptomatic and untreated brain metastasis is an exclusion criterion in all clinical trials [20]. CHECKMATE 204 is the only immunotherapy-based trial with a long-term follow-up [50]. In this trial, patients with symptomatic brain metastasis had the worst outcome (OS less than 6 months), which was attributed to the use of high doses of corticosteroids [52]. High doses of corticosteroids interfere with the activity of ICI and present a unique challenge in managing patients with brain metastasis from melanoma [52]. In such a scenario, chemotherapy can serve as a bridge to switching to immunotherapy or to an immunotherapy-based trial, as steroids do not impair chemotherapy activity. We had a similar experience with several of our patients receiving mVCT, where dexamethasone was used at various intervals to control the edema in the brain.

In our experience, mVCT also appears to control intracranial metastasis better. Previous experience with chemotherapy for melanoma-associated brain metastasis dated from the pre-IO era mostly showed dismal responses. The combination of single-agent fotemustine (a nitrosourea) with radiation showed only a 16% ORR and a median time to progression of 56 days [53]. Although TMZ was superior to DTIC in prolonging PFS with better health-related quality of life [45], median PFS for TMZ monotherapy was 2.4 to 5 months [51,54,55]. A phase II trial combining TMZ with lomustine showed no responses [56]. In our study, 8 out of 10 patients had brain metastasis upon progression after previous therapies. The median ICPFS with mVCT was 7.5 months, with an ICRR of 62.5% and an ICCBR of 100%, suggesting that mVCT is an effective regimen in patients with RRM and brain metastasis who are not eligible for clinical trials. This is a unique feature of our study, as the HECOG Phase II trial did not include patients with brain metastasis [44].

This better response than previous reports is probably because cisplatin has been shown to attenuate MGMT activity [57,58,59], which is responsible for acquired tumor resistance to TMZ, thereby enhancing its antitumor activity when combined [48]. In addition, the ICI that all our patients received prior to the mVCT might sensitize tumor cells by removing inhibitory signals of T-cell activation and promoting infiltration of immune cells into the tumor [60]. Chemotherapy could potentiate these effects by debulking the primary tumor, thereby further inhibiting tumor evasion and directly stimulating the immune cells already infiltrating the tumor [61]. Consistently, many recent studies have reported good outcomes with chemotherapy following ICI in other types of cancers [62,63,64,65,66]. Although single-cell RNA sequencing has led to a better interpretation of immune cells and their interaction with tumor/stromal cells in the tumor microenvironment, shaping the spatial landscape of anti-tumor immunity and understanding its relationship with intratumor heterogeneity of cancer cells will be our next challenge to decipher the mechanisms of ICI, define biomarkers, and identify key therapeutic targets [67].

### 4.2. Identifying Barriers to Tumor Infiltrating Lymphocyte Therapy and Clinical Trials

The patients in our cohort received mVCT before TILs became available at our institution. However, our institution offers several clinical trials, and yet these patients were found ineligible. Some of these patients were referred to other institutions for TIL therapy; however, they faced several challenges along the way. Although TILs and clinical trial enrollment are recognized steps in the journey of a patient with RRM, several challenges remain in enrolling patients in either program.

#### 4.2.1. Limitations of Tumor Infiltrating Lymphocyte Therapy

Adoptive cell therapy (ACT), the umbrella term for TIL therapy, has been in development since 1986 [68,69]. The approval of checkpoint inhibitors in the early 21st century slowed ACT development for a while, until the need to develop treatment for IO-refractory cancers became apparent by mid-2010. The first approval for TIL therapy came in February 2024, when the USFDA approved Lifileucel^®^, based on the results of the phase 2 C-144-01 trial [70]. At 5-year follow-up, the ORR was 31.4%, with 5.9% of patients achieving a CR and 25.5% a PR [71]. The 5-year OS was 19.7%, and the median OS was 13.9 months [71]. To date, Lifileucel^®^ is the first and only ACT-based, US FDA-approved therapy in the treatment-refractory setting for patients with melanoma who have progressed on an anti-PD1 agent and BRAF/MEK-I (in those with BRAF V600E/K-positive melanoma) [70]. NCCN also recognizes escalation to dual checkpoint inhibitors, intratumoral TVEC injections (in specific circumstances), and the combination of pembrolizumab (anti-PD1) and lenvatinib (VEGF-TKI) as IO-based treatments in RRM; however, they are not USFDA-approved specifically for the relapsed-refractory setting [4].

Despite the success of ACT with Lifileucel^®^, several limitations remain. Access to TIL therapy is challenging for many patients, especially those managed outside an IOVANCE-approved center (such as community oncology or a non-IOVANCE-approved cancer center) [29,72,73,74]. Lifileucel^®^ based TIL therapy is only available at centers approved by IOVANCE [29,73,74]. This helps identify appropriate multidisciplinary teams, coordinate tissue collection, and maintain the standard of care for delivering TIL therapy [29,74]. Delivering TIL therapy requires coordination among surgical oncology (tumor harvest), cell therapy teams (lymphocyte depletion therapy and administration of high-dose interleukin-2), and medical oncology (bridging therapy, post-TIL monitoring, and management of immunotherapy-related adverse events) [29,72,73,74,75]. As with any other cell therapy product, obtaining third-party approvals and managing inpatient care require enormous resources [29,72,73,74]. Even with coordinated care, delays remain in initial consultations with the cell therapy and surgical teams and in scheduling the tumor harvest date, which must be coordinated with IOVANCE [72,73,74,75].

Not all patients with melanoma experiencing progression on ICI or BRAF/MEK-I are offered TIL therapy. Stringent criteria are followed to select the most appropriate patients [29,75]. Patients with poor performance status, rapidly progressing disease, or symptomatic or active brain metastasis are not considered appropriate for TIL therapy. Selection of an appropriate location for tumor harvest and removing at least a 1.5 cm tumor are surgical barriers to TIL therapy [74,76]. In addition, the final product must meet specific requirements set by the USFDA (a cell dose between 7.5 × 10^9^ and 72 × 10^9^ viable cells) [70]. The finalized TIL product may be out of specification in up to 10–28.5% of patients [72,73]. Despite meeting all the above requirements, several patients would not receive TILs due to rapid progression of the melanoma in the bridging time period, which leads to rapid decline in the performance status, organ damage, or death of the patient [29,72,73]. Depending on the study, the time from referral to TIL infusion averages 106–128 days in IOVANCE-approved centers [72,73]. In a rapidly progressing disease, this unintended delay in treatment proves fatal for several patients. Hence, there is a need for an effective bridging therapy to prevent progression, especially after tumor harvest is complete.

#### 4.2.2. Can mVCT Serve as a Bridging Therapy?

Given its modest toxicity and reasonable efficacy (especially intracranial efficacy), mVCT may appear to be a reasonable bridging therapy. The effect of TMZ on the tumor microenvironment (TME) has been extensively studied in brain tumors (glioblastoma and high-grade gliomas), as numerous attempts have been made to convert these ‘immune-cold’ tumors into ‘immune-hot’ tumors [77]. Although TMZ does not seem to be an effective adjunct to ICI in brain tumors, in melanoma, the addition of high-dose TMZ to Ipilimumab yielded favorable results in the pre-doublet immunotherapy era [78]. The combination of ipilimumab 10 mg/kg and TMZ 200 mg/m^2^/day (Day 1–4) yielded an ORR of ~30%, which was higher than Ipilimumab 10 mg/kg monotherapy [78]. The authors attributed this benefit to preferential killing of T-regulator cells, rather than the T-effector cells, although no correlative studies were done during the trial [78,79,80]. Small case series have elucidated the benefit of low-dose metronomic TMZ (75 mg/m^2^/day for 6 weeks in an 8-week cycle) [81,82]. This clinical observation aligns with earlier studies in which a low dose of TMZ administered before a dendritic cell vaccine reduced the population of circulating Foxp3+ Tregs by 60%, thereby improving antigen presentation and immune response [79]. Overall, TMZ-based combination chemotherapy to bridge tumor harvest and TIL receipt may be a favorable option. However, more studies are needed before wider adoption of mVCT in this regard.

#### 4.2.3. Principal Reasons for Clinical Trial Ineligibility

National guidelines for any malignancy list clinical trial enrollment as a top priority. Clinical trials not only improve the efficacy of existing treatments but also offer patients options beyond approved medicines [83,84]. However, there are several barriers to clinical trial enrollment. It is estimated that only 2–3% of adult patients participate in clinical trials, despite Americans viewing trial participation favorably [85,86,87]. Unavailability of the trial at the treating center, patient ineligibility despite the trial being available, and refusal to enroll are among the top barriers to clinical trial enrollment [83]. Beyond institutional barriers, eligibility criteria also limit enrollment, especially for trials evaluating immunotherapies [18,88]. Active or untreated brain metastasis, poor performance status, organ dysfunction, active treatment for a concurrent malignancy, or receiving treatment for another malignancy within the last five years are some of the prominent reasons for excluding patients from trials [19,20,89]. With the success of immunotherapy in melanoma, trials have increasingly focused on including checkpoint inhibitors or other forms of immunotherapy [90]. This creates additional challenges in recruitment. Patients with existing autoimmune disease, or those requiring immunosuppressants for any reason (transplant status, autoimmune condition, etc.), are excluded [91].

Socioeconomic factors play a major role in clinical trial enrollment. Distance from the trial center, third-party coverage, and literacy status are important barriers to clinical trial enrollment [87]. Patients with incomes below $50,000 were 32% less likely to participate in a clinical trial, with participation decreasing across lower-income subgroups [92]. Only 41% of insurance plans had networks that included NCI-funded cancer centers [93], and concern about insurance denial is a reason for 8% of eligible patients to decline trials [93,94,95]. Traveling to a tertiary care center to participate in a trial is a significant financial burden for patients [96,97]. A longer distance from the trial center is a recognized barrier to clinical trial enrollment [98]. In patients with metastatic melanoma, rural zip codes are linked to poor trial access, and distance from a cancer center is the second most common reason for trial-eligible patients to decline participation in a clinical trial [94,99]. In addition, phase 1 trials are associated with frequent and longer hospital visits, which are associated with additional financial toxicity for the patient, especially those living far from the trial center [100,101]. Demographic factors are also a barrier to clinical trial enrollment. Black or Hispanic patients with cancer have lower participation rates relative to white patients across multiple oncology trials [102]. This group of patients is more likely to be treated at community practices with little or no access to trials [103]. In our experience, inability to speak, read, or write English is also a barrier to clinical trial enrollment [104]. Small studies indicate that patients treated by bilingual physicians are more likely to enroll in clinical trials [105]. For patients who do not speak English as their native language (mostly minorities), inability to understand the consent process and general mistrust of research are major barriers to clinical trial enrollment [106]. In our cohort, several patients had one or the other issue that prevented clinical trial enrollment, hence forcing us to choose a cytotoxic chemotherapy-based option (Figure 1).

## 5. Conclusions

This manuscript demonstrates that mVCT is a safe and effective regimen for patients with RRM and active brain metastasis who are not eligible for clinical trials, either because of physical conditions (brain metastasis, requirement of continuous immunosuppression, etc.) or social determinants. In such patients, the mVCT regimen can serve as an effective salvage regimen and a bridge till a clinical trial becomes available. We also identify social issues as significant barriers to trial enrollment alongside ICI-related toxicities. This highlights the need for future investigations to improve patient access to clinical trials and, in turn, clinical outcomes.

## Figures and Tables

**Table 1:** Baseline characteristics of the study population and response/toxicity of mVCT regimen.

Pt	Age/ Sex	Race	Type of Melanoma	Previous Therapies	Stage at Starting CVT	Hx. of GKRS	Number of CVT Cycles Received	Overall Response	Intracranial Response	PFS (Months)	ICPFS (Months)	Toxicity
1	59/M	White	Cutaneous	Pembrolizumab (neoadjuvant) Ipilimumab + Nivolumab	4 (M1d)	Yes	6	CR	PR	7	7	G2 Fatigue
2	91/M	White	Cutaneous	Ipilimumab + Nivolumab	4 (M1d)	No	6	PR	CR	12	15	* G4 Fatigue
3	48/F	White	Mucosal	Pembrolizumab, Ipilimumab + Nivolumab	4 (M1d)	No	6	PR	CR	10	12	G3 myelosuppression
4	72/M	White	Mucosal	Pembrolizumab, Carbo/Paclitaxel, PARPi	4 (M1d)	Yes	6	PR	SD	7	9	G2 Fatigue
5	49/F	White	Cutaneous	BRAFi + MEKi, Ipilimumab + Nivolumab	4 (M1d)	Yes	3	SD	SD	3	6	* G4 Myelosuppression
6	51/F	Black	Acral	Ipilimumab + Nivolumab	4 (M1d)	Yes	7	SD	PR	7	8	G2 Fatigue
7	70/M	White	Acral	Nivolumab, TVECs, MEKi	4 (M1d)	Yes	3	PD	SD	1	6	None
8	41/M	White	Cutaneous	Interferon, Ipilimumab + Nivolumab BRAFi + MEKi, PEG-IL12, Pembrolizumab + Lenvatinib, Carbo/Paclitaxel	4 (M1d)	Yes	2	PD	PR	0	6	G1 Fatigue
9	49/M	White	Acral	Pembrolizumab, Ipilimumab + Nivolumab	4 (M1b)	No	3	PD	N/A	1	N/A	G2 Fatigue
10	72/F	White	Cutaneous	Ipilimumab + Nivolumab	3 LAUR	No	2	PR	N/A	10	N/A	** G3 Fatigue

* These patients required a dose reduction in chemotherapy due to the side effects. ** The toxicity led to discontinuation of Cisplatin and Vinblastine, but the patient tolerated 10 cycles of Temozolomide in combination with Pembrolizumab afterward, followed by Pembrolizumab monotherapy, and is currently in CR. Abbreviations: Pt—patient; TVEC—talimogene laherparepvec; PFS—progression-free survival; LAUR—locally advanced unresectable; N/A—not applicable.

**Table 2:** BRAF mutational status, post-mVCT treatment course, and survival outcomes.

No.	Stage at Presentation	BRAF Mutational Status	Previous Treatment	Stage at Starting mVCT	Post-mVCT Treatments	Survival Status	OS (Months)
1	Stage III (Clinical)	Wild type	Pembrolizumab (neoadjuvant) → Progression → I3 + N1	Stage IV (M1d)	Laparoscopic excision of hepatic artery lymph node and GKRS/craniotomy with tumor resection for brain disease. Expired due to progression of brain disease	Dead	17
2	Stage III (LAUR)	Wild type	I1 + N3	Stage IV (M1d)	Declined further surveillance/treatment; lost to follow-up after last contact at 18 months	Alive	18
3	Stage IVA (Mucosal)	Wild type	Adjuvant Pembrolizumab → Relapse (M1d) → I3 + N1 → Grade 4 pneumonitis	Stage IV (M1d)	Pembrolizumab → G3 pneumonitis → mVCT rechallenge × 6 cycles → SD → Olaparib for HRD signature positive at relapse → Did not tolerate → Recently received Lifileucel	Alive	32
4	Stage III (Mucosal)	Wild type	Adjuvant Pembrolizumab → Grade 4 hepatitis	Stage IV (M1d)	Completed 2 years of Pembrolizumab → Currently CR on surveillance	Alive	35
5	Stage IV (M1d)— Visceral crises	BRAF V600E	Encorafenib + Binimetinib → I3 + N1 → Grade 4 hepatitis	Stage IV (M1d)	Expired due to rapid progression of disease	Dead	6
6	Stage IIC → M1d (Acral)	Non-BRAF V600E/K	Clinical trial (I3 + N1) → Grade 3 dermatitis, Grade 3 anemia (PRCA and AIHA)	Stage IV (M1d)	GKRS(s) → Currently on Encorafenib + Binimetinib	Alive	37
7	Stage IIID → LAUR (Acral)	Wild type	Adjuvant Nivolumab → Grade 3 colitis, encephalitis	Stage IV (M1d)	Received TIL therapy in a phase 1 trial but expired due to progression of disease	Dead	8
8	Stage IIIB → M1b	BRAF V600E	Adjuvant Interferon → BRAF/MEK-I → 3 Phase 1 clinical trials → Pembrolizumab + Lenvatinib	Stage IV (M1d)	Expired due to rapid progression of disease	Dead	5
9	Stage IIIC → M1b (Acral)	Non-BRAF V600E/K	Adjuvant pembrolizumab → Progression → I3 + N1 → Grade 4 colitis	Stage IV (M1b)	Enrolled in Phase 1 Pan-RAF inhibitor trial → Enrolled in TIL therapy but harvest failed → Expired due to progression of disease	Dead	22
10	Stage III (LAUR)- in-transit metastasis	Wild type	I1 + N3	Stage III (LAUR)	Switched to Temozolomide + Pembrolizumab → Pembrolizumab maintenance → Currently CR on surveillance	Alive	25

Abbreviations: I3 + N1—Ipilimumab 3 mg/kg and Nivolumab 1mg/kg; I1 + N3—Ipilimumab 1 mg/kg and Nivolumab 3mg/kg; PRCA—pure red cell aplasia; AIHA—autoimmune hemolytic anemia; LAUR—Locally advanced unresectable; GKRS—Gamma Knife Radiosurgery; TIL—tumor-infiltrating lymphocyte; HRD—homologous recombination deficiency.

## Data Availability

Data will be available from the corresponding author upon request.

## Abbreviations

The following abbreviations are used in this manuscript:

ACT	Adoptive cell therapy
BRAF	B-Raf proto-oncogene, serine/threonine kinase
BRAF/MEK-I	BRAF and MEK inhibitors
CAR-T	Chimeric antigen receptor T-cell therapy
CBR	Clinical benefit rate
CR	Complete response
CTLA-4	Cytotoxic T-lymphocyte-associated protein 4
CYP450	Cytochrome P450
DNA	Deoxyribonucleic acid
DTIC	Dacarbazine
ECOG	Eastern Cooperative Oncology Group
EFS	Event-free survival
ERBB2	Erb-B2 receptor tyrosine kinase 2
FDA	Food and Drug Administration
Foxp3	Forkhead box P3
GKRS	Gamma-knife radiosurgery
HECOG	Hellenic Cooperative Oncology Group
IC	Intracranial
ICCBR	Intracranial clinical benefit rate
ICI	Immune checkpoint inhibitor
ICPFS	Intracranial progression-free survival
ICRR	Intracranial response rate
IO	Immunotherapy
IQR	Interquartile range
IRAE	Immunotherapy-related adverse event
M1b	AJCC stage M1b melanoma (lung metastasis)
M1d	AJCC stage M1d melanoma (CNS metastasis)
MDACC	MD Anderson Cancer Center
MEK	Mitogen-activated extracellular signal-regulated kinase
MGMT	O6-methylguanine–DNA methyltransferase
MTIC	3-methyl-(triazen-1-yl)imidazole-4-carboximid e
mVCT	Modified Vinblastine–Cisplatin–Temozolomide
NCCN	National Comprehensive Cancer Network
NTRK	Neurotrophic tyrosine receptor kinase
ORR	Overall response rate
OS	Overall survival
PARP	Poly ADP-ribose polymerase
PD	Progressive disease
PD-1	Programmed cell death protein 1
PFS	Progression-free survival rate
PR	Partial response
RCT	Randomized controlled trial
RECIST	Response Evaluation Criteria in Solid Tumors
RET	Rearranged during transfection
RRM	Relapsed-refractory melanoma
SD	Stable disease
SLNB	Sentinel lymph node biopsy
SPSS	Statistical Package for the Social Sciences
TCR	T-cell receptor
TIL	Tumor-infiltrating lymphocyte
TKI	Tyrosine kinase inhibitor
TME	Tumor microenvironment
TMZ	Temozolomide
Tregs	Regulatory T cells
T-VEC	Talimogene laherparepvec
VCD	Vinblastine–Cisplatin–Dacarbazine
VCT	Vinblastine–Cisplatin–Temozolomide
VEGF-TKI	Vascular endothelial growth factor tyrosine kinase inhibitor
WLE	Wide local excision

## References

[1] Saginala K., Barsouk A., Aluru J.S., Rawla P., Barsouk A. (2021). Epidemiology of Melanoma. Med. Sci..

[2] Joshi U.M., Kashani-Sabet M., Kirkwood J.M. (2025). Cutaneous Melanoma: A Review. JAMA.

[3] Siegel R.L., Kratzer T.B., Wagle N.S., Sung H., Jemal A. (2026). Cancer statistics, 2026. CA A Cancer J. Clin..

[4] NCCN Melanoma: Cutaneous. https://www.nccn.org/professionals/physician_gls/pdf/cutaneous_melanoma.pdf.

[5] Patel S.P., Othus M., Chen Y., Wright G.P., Yost K.J., Hyngstrom J.R., Hu-Lieskovan S., Lao C.D., Fecher L.A., Truong T.-G. (2023). Neoadjuvant–Adjuvant or Adjuvant-Only Pembrolizumab in Advanced Melanoma. N. Engl. J. Med..

[6] Blank C.U., Lucas M.W., Scolyer R.A., Wiel B.A.v.d., Menzies A.M., Lopez-Yurda M., Hoeijmakers L.L., Saw R.P.M., Lijnsvelt J.M., Maher N.G. (2024). Neoadjuvant Nivolumab and Ipilimumab in Resectable Stage III Melanoma. N. Engl. J. Med..

[7] Tawbi H.A., Schadendorf D., Lipson E.J., Ascierto P.A., Matamala L., Castillo Gutiérrez E., Rutkowski P., Gogas H.J., Lao C.D., De Menezes J.J. (2022). Relatlimab and Nivolumab versus Nivolumab in Untreated Advanced Melanoma. N. Engl. J. Med..

[8] Wolchok J.D., Chiarion-Sileni V., Gonzalez R., Grob J.-J., Rutkowski P., Lao C.D., Cowey C.L., Schadendorf D., Wagstaff J., Dummer R. (2022). Long-Term Outcomes with Nivolumab Plus Ipilimumab or Nivolumab Alone Versus Ipilimumab in Patients with Advanced Melanoma. J. Clin. Oncol..

[9] Garg A., Rao R., Tejawinata F., Shamita G.A.N., Herpel M.S., Yoshida A., Goolamier G., Sidiropoulos J., Sheng I.Y., Abboud S.T. (2025). Advances in Oncolytic Viral Therapy in Melanoma: A Comprehensive Review. Vaccines.

[10] Shet P., Ghosh P., Varshney K., Sawdagar S. (2026). Chimeric Antigen Receptor T-Cell Therapy for the Treatment of Melanoma: A Systematic Review of Phase One Clinical Trials. Technol. Cancer Res. Treat..

[11] Sun Q., Zhang X., Wang L., Gao X., Xiong Y., Liu L., Wei F., Yang L., Ren X. (2019). T-cell receptor gene therapy targeting melanoma-associated antigen-A4 by silencing of endogenous TCR inhibits tumor growth in mice and human. Cell Death Dis..

[12] Wermke M., Araujo D.M., Chatterjee M., Tsimberidou A.M., Holderried T.A.W., Jazaeri A.A., Reshef R., Bokemeyer C., Alsdorf W., Wetzko K. (2025). Autologous T cell therapy for PRAME^+^ advanced solid tumors in HLA-A*02^+^ patients: A phase 1 trial. Nat. Med..

[13] Ma V.T., Steimle A.K., Patel J.D., Burkey C., Moser J.C., Javed A., tul Rehman H., Seker M.E., Birbrair A., Ozkan O.S. (2026). Updates on intratumoral therapies in melanoma. Cancer.

[14] Subbiah V. (2025). Tissue-agnostic cancer therapies: Promise, reality, and the path forward. Nat. Commun..

[15] Long G.V., Atkinson V., Ascierto P.A., Brady B., Dutriaux C., Maio M., Mortier L., Hassel J.C., Rutkowski P., McNeil C. (2015). Nivolumab improved survival vs dacarbazine in patients with untreated advanced melanoma. J. Transl. Med..

[16] Maio M., Grob J.J., Aamdal S., Bondarenko I., Robert C., Thomas L., Garbe C., Chiarion-Sileni V., Testori A., Chen T.T. (2015). Five-year survival rates for treatment-naive patients with advanced melanoma who received ipilimumab plus dacarbazine in a phase III trial. J. Clin. Oncol..

[17] Larkin J., Minor D., D’Angelo S., Neyns B., Smylie M., Miller W.H., Gutzmer R., Linette G., Chmielowski B., Lao C.D. (2018). Overall Survival in Patients with Advanced Melanoma Who Received Nivolumab Versus Investigator’s Choice Chemotherapy in CheckMate 037: A Randomized, Controlled, Open-Label Phase III Trial. J. Clin. Oncol..

[18] Kim E.S., Uldrick T.S., Schenkel C., Bruinooge S.S., Harvey R.D., Magnuson A., Spira A., Wade J.L., Stewart M.D., Vega D.M. (2021). Continuing to Broaden Eligibility Criteria to Make Clinical Trials More Representative and Inclusive: ASCO-Friends of Cancer Research Joint Research Statement. Clin. Cancer Res..

[19] Lichtman S.M., Harvey R.D., Smit M.-A.D., Rahman A., Thompson M.A., Roach N., Schenkel C., Bruinooge S.S., Cortazar P., Walker D. (2017). Modernizing Clinical Trial Eligibility Criteria: Recommendations of the American Society of Clinical Oncology–Friends of Cancer Research Organ Dysfunction, Prior or Concurrent Malignancy, and Comorbidities Working Group. J. Clin. Oncol..

[20] Lin N.U., Prowell T., Tan A.R., Kozak M., Rosen O., Amiri-Kordestani L., White J., Sul J., Perkins L., Beal K. (2017). Modernizing Clinical Trial Eligibility Criteria: Recommendations of the American Society of Clinical Oncology–Friends of Cancer Research Brain Metastases Working Group. J. Clin. Oncol..

[21] Minasian L.M., Unger J.M. (2020). What Keeps Patients Out of Clinical Trials?. JCO Oncol. Pract..

[22] Nguyen K., Hignett E., Khachemoune A. (2020). Current and emerging treatment options for metastatic melanoma: A focused review. Dermatol. Online J..

[23] Lim S.Y., Shklovskaya E., Lee J.H., Pedersen B., Stewart A., Ming Z., Irvine M., Shivalingam B., Saw R.P.M., Menzies A.M. (2023). The molecular and functional landscape of resistance to immune checkpoint blockade in melanoma. Nat. Commun..

[24] Ascierto P.A., Dreno B., Larkin J., Ribas A., Liszkay G., Maio M., Mandala M., Demidov L., Stroyakovskiy D., Thomas L. (2021). 5-Year Outcomes with Cobimetinib plus Vemurafenib in BRAFV600 Mutation-Positive Advanced Melanoma: Extended Follow-up of the coBRIM Study. Clin. Cancer Res..

[25] Dummer R., Ascierto P.A., Gogas H.J., Arance A., Mandala M., Liszkay G., Garbe C., Schadendorf D., Krajsova I., Gutzmer R. (2018). Encorafenib plus binimetinib versus vemurafenib or encorafenib in patients with BRAF-mutant melanoma (COLUMBUS): A multicentre, open-label, randomised phase 3 trial. Lancet Oncol..

[26] Flaherty K.T., Infante J.R., Daud A., Gonzalez R., Kefford R.F., Sosman J., Hamid O., Schuchter L., Cebon J., Ibrahim N. (2012). Combined BRAF and MEK inhibition in melanoma with BRAF V600 mutations. N. Engl. J. Med..

[27] Schadendorf D., Dummer R., Flaherty K.T., Robert C., Arance A., de Groot J.W.B., Garbe C., Gogas H.J., Gutzmer R., Krajsova I. (2024). COLUMBUS 7-year update: A randomized, open-label, phase III trial of encorafenib plus binimetinib versus vemurafenib or encorafenib in patients with BRAF V600E/K-mutant melanoma. Eur. J. Cancer.

[28] Priantti J.N., Vilbert M., Madeira T., Moraes F.C.A., Hein E.C.K., Saeed A., Cavalcante L. (2023). Efficacy and Safety of Rechallenge with BRAF/MEK Inhibitors in Advanced Melanoma Patients: A Systematic Review and Meta-Analysis. Cancers.

[29] Allison Betof W., Omid H., Krishna K., Rodabe A., Marcus O.B., John H., Sarah N., Igor P., Amod S., Michael R.B. (2024). Expert consensus guidelines on management and best practices for tumor-infiltrating lymphocyte cell therapy. J. Immunother. Cancer.

[30] Saint-Jean M., Fronteau C., Peuvrel L., Khammari A., Varey E., Quereux G., Dreno B. (2020). Chemotherapy efficacy after first-line immunotherapy in 18 advanced melanoma patients. Medicine.

[31] Mangin M.A., Boespflug A., Maucort Boulch D., Vacheron C.H., Carpentier I., Thomas L., Dalle S. (2021). Decreased survival in patients treated by chemotherapy after targeted therapy compared to immunotherapy in metastatic melanoma. Cancer Med..

[32] Karachaliou G.S., Ayvali F., Collichio F.A., Lee C.B., Ivanova A., Ollila D.W., Moschos S.J. (2020). Chemotherapy Following PD-1 Inhibitor Blockade in Patients with Unresectable Stage III/Stage IV Metastatic Melanoma: A Single Academic Institution Experience. Oncology.

[33] Wang X., Xu W., Chi Z., Si L., Sheng X., Kong Y., Zhou L., Mao L., Lian B., Tang B. (2021). Chemotherapy combined with antiangiogenic drugs as salvage therapy in advanced melanoma patients progressing on PD-1 immunotherapy. Transl. Oncol..

[34] Vera Aguilera J., Paludo J., McWilliams R.R., Zhang H., Li Y., Kumar A.B., Failing J., Kottschade L.A., Block M.S., Markovic S.N. (2020). Chemo-immunotherapy combination after PD-1 inhibitor failure improves clinical outcomes in metastatic melanoma patients. Melanoma Res..

[35] Stagno A., Vari S., Annovazzi A., Anelli V., Russillo M., Cognetti F., Ferraresi V. (2021). Case Report: Rechallenge with BRAF and MEK Inhibitors in Metastatic Melanoma: A Further Therapeutic Option in Salvage Setting?. Front. Oncol..

[36] Tietze J.K., Forschner A., Loquai C., Mitzel-Rink H., Zimmer L., Meiss F., Rafei-Shamsabadi D., Utikal J., Bergmann M., Meier F. (2018). The efficacy of re-challenge with BRAF inhibitors after previous progression to BRAF inhibitors in melanoma: A retrospective multicenter study. Oncotarget.

[37] Cybulska-Stopa B., Rogala P., Czarnecka A.M., Galus L., Dziura R., Rajczykowski M., Kubiatowski T., Wisniewska M., Gega-Czarnota A., Teterycz P. (2020). BRAF and MEK inhibitors rechallenge as effective treatment for patients with metastatic melanoma. Melanoma Res..

[38] Legha S.S., Ring S., Papadopoulos N., Plager C., Chawla S., Benjamin R. (1989). A prospective evaluation of a triple-drug regimen containing cisplatin, vinblastine, and dacarbazine (CVD) for metastatic melanoma. Cancer.

[39] Atkins M.B., Hsu J., Lee S., Cohen G.I., Flaherty L.E., Sosman J.A., Sondak V.K., Kirkwood J.M. (2008). Phase III trial comparing concurrent biochemotherapy with cisplatin, vinblastine, dacarbazine, interleukin-2, and interferon alfa-2b with cisplatin, vinblastine, and dacarbazine alone in patients with metastatic malignant melanoma (E3695): A trial coordinated by the Eastern Cooperative Oncology Group. J. Clin. Oncol..

[40] Legha S.S., Ring S., Bedikian A., Plager C., Eton O., Buzaid A.C., Papadopoulos N. (1996). Treatment of metastatic melanoma with combined chemotherapy containing cisplatin, vinblastine and dacarbazine (CVD) and biotherapy using interleukin-2 and interferon-alpha. Ann. Oncol..

[41] Ron I.G., Sarid D., Ryvo L., Sapir E.E., Schneebaum S., Metser U., Asna N., Inbar M.J., Safra T. (2006). A biochemotherapy regimen with concurrent administration of cisplatin, vinblastine, temozolomide (Temodal), interferon-alfa and interleukin-2 for metastatic melanoma: A phase II study. Melanoma Res..

[42] Bafaloukos D., Tsoutsos D., Kalofonos H., Chalkidou S., Panagiotou P., Linardou E., Briassoulis E., Efstathiou E., Polyzos A., Fountzilas G. (2005). Temozolomide and cisplatin versus temozolomide in patients with advanced melanoma: A randomized phase II study of the Hellenic Cooperative Oncology Group. Ann. Oncol..

[43] Cao M.G., Malvehy J., Martí R., Conill C., Sánchez M., Martín M., Carrera C., Herrero J., Gascón P., Mellado B. (2006). Biochemotherapy with temozolomide, cisplatin, vinblastine, subcutaneous interleukin-2 and interferon-α in patients with metastatic melanoma. Melanoma Res..

[44] Linardou H., Pentheroudakis G., Varthalitis I., Gogas H., Pectasides D., Makatsoris T., Fountzilas G., Bafaloukos D. (2015). Predictive biomarkers to chemotherapy in patients with advanced melanoma receiving the combination of cisplatin--vinblastine--temozolomide (PVT) as first-line treatment: A study of the Hellenic Cooperative Oncology Group (HECOG). Anticancer Res..

[45] Middleton M.R., Grob J.J., Aaronson N., Fierlbeck G., Tilgen W., Seiter S., Gore M., Aamdal S., Cebon J., Coates A. (2000). Randomized phase III study of temozolomide versus dacarbazine in the treatment of patients with advanced metastatic malignant melanoma. J. Clin. Oncol..

[46] Amirmostofian M., Pourahmad Jaktaji J., Soleimani Z., Tabib K., Tanbakosazan F., Omrani M., Kobarfard F. (2013). Synthesis and Molecular-cellular Mechanistic Study of Pyridine Derivative of Dacarbazine. Iran. J. Pharm. Res..

[47] Zhang J., Stevens M.F., Bradshaw T.D. (2012). Temozolomide: Mechanisms of action, repair and resistance. Curr. Mol. Pharmacol..

[48] Hegi M.E., Diserens A.C., Gorlia T., Hamou M.F., de Tribolet N., Weller M., Kros J.M., Hainfellner J.A., Mason W., Mariani L. (2005). MGMT gene silencing and benefit from temozolomide in glioblastoma. N. Engl. J. Med..

[49] Brada M., Judson I., Beale P., Moore S., Reidenberg P., Statkevich P., Dugan M., Batra V., Cutler D. (1999). Phase I dose-escalation and pharmacokinetic study of temozolomide (SCH 52365) for refractory or relapsing malignancies. Br. J. Cancer.

[50] Tawbi H.A., Forsyth P.A., Algazi A., Hamid O., Hodi F.S., Moschos S.J., Khushalani N.I., Lewis K., Lao C.D., Postow M.A. (2018). Combined Nivolumab and Ipilimumab in Melanoma Metastatic to the Brain. N. Engl. J. Med..

[51] Agarwala S.S., Kirkwood J.M., Gore M., Dreno B., Thatcher N., Czarnetski B., Atkins M., Buzaid A., Skarlos D., Rankin E.M. (2004). Temozolomide for the treatment of brain metastases associated with metastatic melanoma: A phase II study. J. Clin. Oncol..

[52] Tawbi H.A., Forsyth P.A., Hodi F.S., Algazi A.P., Hamid O., Lao C.D., Moschos S.J., Atkins M.B., Lewis K., Postow M.A. (2021). Long-term outcomes of patients with active melanoma brain metastases treated with combination nivolumab plus ipilimumab (CheckMate 204): Final results of an open-label, multicentre, phase 2 study. Lancet Oncol..

[53] Mornex F., Thomas L., Mohr P., Hauschild A., Delaunay M.M., Lesimple T., Tilgen W., Bui B.N., Guillot B., Ulrich J. (2003). A prospective randomized multicentre phase III trial of fotemustine plus whole brain irradiation versus fotemustine alone in cerebral metastases of malignant melanoma. Melanoma Res..

[54] Hofmann M., Kiecker F., Wurm R., Schlenger L., Budach V., Sterry W., Trefzer U. (2006). Temozolomide with or without radiotherapy in melanoma with unresectable brain metastases. J. Neurooncol..

[55] Margolin K., Atkins B., Thompson A., Ernstoff S., Weber J., Flaherty L., Clark I., Weiss G., Sosman J., Smith J. (2002). Temozolomide and whole brain irradiation in melanoma metastatic to the brain: A phase II trial of the Cytokine Working Group. J. Cancer Res. Clin. Oncol..

[56] Larkin J.M., Hughes S.A., Beirne D.A., Patel P.M., Gibbens I.M., Bate S.C., Thomas K., Eisen T.G., Gore M.E. (2007). A phase I/II study of lomustine and temozolomide in patients with cerebral metastases from malignant melanoma. Br. J. Cancer.

[57] Piccioni D., D’atri S., Papa G., Caravita T., Franchi A., Bonmassar E., Graziani G. (1995). Cisplatin increases sensitivity of human leukemic blasts to triazene compounds. J. Chemother..

[58] D’Atri S., Graziani G., Lacal P.M., Nisticò V., Gilberti S., Faraoni I., Watson A.J., Bonmassar E., Margison G.P. (2000). Attenuation of O^6^-methylguanine-DNA methyltransferase activity and mRNA levels by cisplatin and temozolomide in Jurkat cells. J. Pharmacol. Exp. Ther..

[59] Wang L.-G., Setlow R.B. (1989). Inactivation of O 6-alkylguanine-DNA alkyltransferase in HeLa cells by cisplatin. Carcinogenesis.

[60] Wei S.C., Duffy C.R., Allison J.P. (2018). Fundamental mechanisms of immune checkpoint blockade therapy. Cancer Discov..

[61] Sordo-Bahamonde C., Lorenzo-Herrero S., Gonzalez-Rodriguez A.P., Martínez-Pérez A., Rodrigo J.P., García-Pedrero J.M., Gonzalez S. (2023). Chemo-immunotherapy: A new trend in cancer treatment. Cancers.

[62] Kato R., Hayashi H., Chiba Y., Miyawaki E., Shimizu J., Ozaki T., Fujimoto D., Toyozawa R., Nakamura A., Kozuki T. (2020). Propensity score–weighted analysis of chemotherapy after PD-1 inhibitors versus chemotherapy alone in patients with non–small cell lung cancer (WJOG10217L). J. Immunother. Cancer.

[63] Bersanelli M., Buti S., Giannarelli D., Leonetti A., Cortellini A., Russo G.L., Signorelli D., Toschi L., Milella M., Pilotto S. (2020). Chemotherapy in non-small cell lung cancer patients after prior immunotherapy: The multicenter retrospective CLARITY study. Lung Cancer.

[64] Park S.E., Lee S.H., Ahn J.S., Ahn M.-J., Park K., Sun J.-M. (2018). Increased response rates to salvage chemotherapy administered after PD-1/PD-L1 inhibitors in patients with non–small cell lung cancer. J. Thorac. Oncol..

[65] Saleh K., Daste A., Martin N., Pons-Tostivint E., Auperin A., Herrera-Gomez R.G., Baste-Rotllan N., Bidault F., Guigay J., Le Tourneau C. (2019). Response to salvage chemotherapy after progression on immune checkpoint inhibitors in patients with recurrent and/or metastatic squamous cell carcinoma of the head and neck. Eur. J. Cancer.

[66] Szabados B., van Dijk N., Tang Y.Z., van der Heijden M.S., Wimalasingham A., de Liano A.G., Chowdhury S., Hughes S., Rudman S., Linch M. (2018). Response rate to chemotherapy after immune checkpoint inhibition in metastatic urothelial cancer. Eur. Urol..

[67] Zhang Y., Zhang Z. (2020). The history and advances in cancer immunotherapy: Understanding the characteristics of tumor-infiltrating immune cells and their therapeutic implications. Cell. Mol. Immunol..

[68] Rosenberg S.A., Spiess P., Lafreniere R. (1986). A new approach to the adoptive immunotherapy of cancer with tumor-infiltrating lymphocytes. Science.

[69] Rosenberg S.A., Packard B.S., Aebersold P.M., Solomon D., Topalian S.L., Toy S.T., Simon P., Lotze M.T., Yang J.C., Seipp C.A. (1988). Use of tumor-infiltrating lymphocytes and interleukin-2 in the immunotherapy of patients with metastatic melanoma. N. Engl. J. Med..

[70] Hu L., Fan C., Bross P., Das A., Cho E.S., Knudson K.M., Tegenge M., Gao Q., Brewer J.R., Theoret M.R. (2025). FDA Approval Summary: Lifileucel for Unresectable or Metastatic Melanoma Previously Treated with an Anti-PD-1-Based Immunotherapy. Clin. Cancer Res..

[71] Medina T., Chesney J.A., Kluger H.M., Hamid O., Whitman E.D., Cusnir M., Thomas S.S., Wermke M., Domingo-Musibay E., Phan G.Q. (2025). Long-Term Efficacy and Safety of Lifileucel Tumor-Infiltrating Lymphocyte Cell Therapy in Patients with Advanced Melanoma: A 5-Year Analysis of the C-144-01 Study. J. Clin. Oncol..

[72] Huynh D., Cabrera G., Crystal J.S., Hernandez-Aya L., Lekakis L.J., Vaccino D., Green D.J., Lutzky J., Wang T.P. (2026). Identifying Factors That Limit Access to Tumor-Infiltrating Lymphocytes (TILs) for Advanced Melanoma at a High Volume Academic Center. Transplant. Cell. Ther..

[73] Desai A., Valenzuela C.D., Christmas K., Lackey D., Trussell J., Hayes-Lattin B., Meyers G., Blackburn M., Kohn A., Stomberg C. (2026). Real-World Clinical Outcomes in Melanoma Patients Treated at a Single-Center Tumor Infiltrating Lymphocyte (TIL) Therapy Program Using Commercial Lifileucel. Transpl. Cell Ther..

[74] Turcotte S., Donia M., Gastman B., Besser M., Brown R., Coukos G., Creelan B., Mullinax J., Sondak V.K., Yang J.C. (2025). Art of TIL immunotherapy: SITC’s perspective on demystifying a complex treatment. J. Immunother. Cancer.

[75] Kottschade L., Rodriguez E.W., Harding S., Ranjan S., McIntyre L., Prieto P.A., Gray L., Joseph J., Swank J. (2025). Tumor-Infiltrating Lymphocyte Cell Therapy for the Treatment of Advanced Melanoma: From Patient Identification to Posttreatment Management. J. Adv. Pract. Oncol..

[76] Mullinax J.E., Egger M.E., McCarter M., Monk B.J., Toloza E.M., Brousseau S., Jagasia M., Sarnaik A. (2022). Surgical considerations for tumor tissue procurement to obtain tumor-infiltrating lymphocytes for adoptive cell therapy. Cancer J..

[77] Hotchkiss K.M., Sampson J.H. (2021). Temozolomide treatment outcomes and immunotherapy efficacy in brain tumor. J. Neurooncol..

[78] Patel S.P., Kim D.W., Bassett R.L., Cain S., Washington E., Hwu W.J., Kim K.B., Papadopoulos N.E., Homsi J., Hwu P. (2017). A phase II study of ipilimumab plus temozolomide in patients with metastatic melanoma. Cancer Immunol. Immunother..

[79] Ridolfi L., Petrini M., Granato A.M., Gentilcore G., Simeone E., Ascierto P.A., Pancisi E., Ancarani V., Fiammenghi L., Guidoboni M. (2013). Low-dose temozolomide before dendritic-cell vaccination reduces (specifically) CD4^+^ CD25^++^ Foxp3^+^ regulatory T-cells in advanced melanoma patients. J. Transl. Med..

[80] Banissi C., Ghiringhelli F., Chen L., Carpentier A.F. (2009). Treg depletion with a low-dose metronomic temozolomide regimen in a rat glioma model. Cancer Immunol. Immunother..

[81] Swami U., Monga V., Freesmeier M., Zhang W., Bossler A.D., Zakharia Y., Milhem M. (2019). Exceptional responses with sequential metronomic temozolomide after pembrolizumab failure in patients with metastatic melanoma. Melanoma Res..

[82] Goodman R.S., Jung S., Quintos J., Johnson D.B. (2023). Therapeutic Responses to Combination Nivolumab and Temozolomide as Salvage Therapy for Metastatic Melanoma: A Case Series. Oncologist.

[83] Unger J.M., Vaidya R., Hershman D.L., Minasian L.M., Fleury M.E. (2019). Systematic Review and Meta-Analysis of the Magnitude of Structural, Clinical, and Physician and Patient Barriers to Cancer Clinical Trial Participation. JNCI J. Natl. Cancer Inst..

[84] Zhang H., Jiang X. (2025). Importance of clinical trials and contributions to contemporary medicine: Commentary. Ann. Med..

[85] Comis R.L., Miller J.D., Aldigé C.R., Krebs L., Stoval E. (2003). Public attitudes toward participation in cancer clinical trials. J. Clin. Oncol..

[86] Giffin R.B., Lebovitz Y., English R.A. (2010). Transforming Clinical Research in the United States: Challenges and Opportunities: Workshop Summary.

[87] Sateren W.B., Trimble E.L., Abrams J., Brawley O., Breen N., Ford L., McCabe M., Kaplan R., Smith M., Ungerleider R. (2002). How sociodemographics, presence of oncology specialists, and hospital cancer programs affect accrual to cancer treatment trials. J. Clin. Oncol..

[88] Stefaniak N., Walker J., Murphy M.L., McKinney M., Liu L., Edge S.B. (2020). Do eligibility criteria restrict access to clinical trials?. J. Clin. Oncol..

[89] Patel R.R., Verma V., Miller A.B., Lin T.A., Jethanandani A., Espinoza A.F., Mainwaring W., Augustyn A., Fuller C.D., Sulman E.P. (2020). Exclusion of patients with brain metastases from cancer clinical trials. Neuro Oncol..

[90] Weiss S.A., Wolchok J.D., Sznol M. (2019). Immunotherapy of Melanoma: Facts and Hopes. Clin. Cancer Res..

[91] Haanen J., Ernstoff M.S., Wang Y., Menzies A.M., Puzanov I., Grivas P., Larkin J., Peters S., Thompson J.A., Obeid M. (2020). Autoimmune diseases and immune-checkpoint inhibitors for cancer therapy: Review of the literature and personalized risk-based prevention strategy. Ann. Oncol..

[92] Unger J.M., Gralow J.R., Albain K.S., Ramsey S.D., Hershman D.L. (2016). Patient Income Level and Cancer Clinical Trial Participation: A Prospective Survey Study. JAMA Oncol..

[93] Kehl K.L., Liao K.P., Krause T.M., Giordano S.H. (2017). Access to Accredited Cancer Hospitals Within Federal Exchange Plans Under the Affordable Care Act. J. Clin. Oncol..

[94] Lara P.N., Higdon R., Lim N., Kwan K., Tanaka M., Lau D.H., Wun T., Welborn J., Meyers F.J., Christensen S. (2001). Prospective evaluation of cancer clinical trial accrual patterns: Identifying potential barriers to enrollment. J. Clin. Oncol..

[95] Chino F., Zafar S.Y. (2019). Financial Toxicity and Equitable Access to Clinical Trials. Am. Soc. Clin. Oncol. Educ. Book.

[96] Nipp R.D., Lee H., Powell E., Birrer N.E., Poles E., Finkelstein D., Winkfield K., Percac-Lima S., Chabner B., Moy B. (2016). Financial burden of cancer clinical trial participation and the impact of a cancer care equity program. Oncologist.

[97] Nipp R.D., Powell E., Chabner B., Moy B. (2015). Recognizing the financial burden of cancer patients in clinical trials. Oncologist.

[98] Ambroggi M., Biasini C., Del Giovane C., Fornari F., Cavanna L. (2015). Distance as a barrier to cancer diagnosis and treatment: Review of the literature. Oncologist.

[99] Mulligan K.M., Zheng D.X., Xu J.R., Cullison C.R., Cwalina T.B., Beveridge M.G., Scott J.F. (2023). Geographic disparities in access to immunotherapy clinical trials for metastatic melanoma. Arch. Dermatol. Res..

[100] Perni S., Moy B., Nipp R.D. (2021). Disparities in phase 1 cancer clinical trial enrollment. Cancer.

[101] Uehara Y., Koyama T., Katsuya Y., Sato J., Sudo K., Kondo S., Yoshida T., Shoji H., Shimoi T., Yonemori K. (2023). Travel Time and Distance and Participation in Precision Oncology Trials at the National Cancer Center Hospital. JAMA Netw. Open.

[102] Pittell H., Calip G.S., Pierre A., Ryals C.A., Altomare I., Royce T.J., Guadamuz J.S. (2023). Racial and Ethnic Inequities in US Oncology Clinical Trial Participation from 2017 to 2022. JAMA Netw. Open.

[103] Altomare I., Wang X., Kaur M., Guadamuz J.S., Falk S., Xiao F., Meropol N.J., Zhao Y. (2024). Are community oncology practices with or without clinical research programs different? A comparison of patient and practice characteristics. JNCI Cancer Spectr..

[104] Muthukumar A.V., Morrell W., Bierer B.E. (2021). Evaluating the frequency of English language requirements in clinical trial eligibility criteria: A systematic analysis using ClinicalTrials.gov. PLoS Med..

[105] Rivelli A., Ozoani-Lohrer O., Lefaiver C., Shields M., Marek A., Robaina M., Fitzpatrick V. (2024). Patient participation in clinical trials conducted by principal investigators who speak one or more language(s) beyond english: Exploring ethnicity as proxy for language. Contemp. Clin. Trials Commun..

[106] Quay T.A.W., Frimer L., Janssen P.A., Lamers Y. (2017). Barriers and facilitators to recruitment of South Asians to health research: A scoping review. BMJ Open.

